# Association of lifestyle and flourishing during the COVID-19 pandemic in Japan

**DOI:** 10.3389/fpsyg.2024.1341711

**Published:** 2024-05-30

**Authors:** Tomoyoshi Shibata, Yui Yamaoka, Nobutoshi Nawa, Hisaaki Nishimura, Yuna Koyama, Jin Kuramochi, Takeo Fujiwara

**Affiliations:** ^1^Department of Public Health, Tokyo Medical and Dental University, Tokyo, Japan; ^2^Interpark Kuramochi Clinic, Utsunomiya, Japan

**Keywords:** flourishing, smoking, drinking, sleep duration, COVID-19, pandemic, well-being

## Abstract

**Introduction:**

COVID-19 have changed our lifestyle and little is known how our lifestyle associated with flourishing during COVID-19. This study examined the association between lifestyle, including sleep time, drinking, and smoking, and flourishing during the COVID-19 pandemic in Japan.

**Methods:**

We used the population-based study, Utsunomiya COVID-19 seROprevalence Neighborhood Association (U-CORONA) survey conducted in November 2021 to examine the association between lifestyle such as sleeping time, drinking and smoking, and flourishing (*n* = 473). Flourishing was assessed with the flourishing index, a 10-item multidimensional scale with five domains. Multivariate linear regression analysis was performed adjusted for sex, age, income, and education.

**Results:**

We found that the flourishing index was significantly lower in the group that slept less than 6 h than in the group that slept 6–8 h (coef = −0.49, SE = 0.17, *p* < 0.01). We also found that drinking once to several times/week showed higher flourishing than those who almost never drink (coef = 0.57, SE = 0.19, *p* < 0.01). Smoking was not associated with flourishing.

**Discussion:**

Sleep duration and drinking habit, but not smoking, may be important for flourishing during the COVID-19 pandemic.

## 1 Introduction

While psychology has focused on mental illness for decades, a newly developed field of positive psychology has started to concern more on the positive side of psychological states, such as well-being (Seligman and Csikszentmihalyi, 2000; Seligman et al., 2005; Ryff, 2022). Moreover, a growing body of literature paid attention to flourishing (Hone et al., 2014; Vanderweele et al., 2020). Vanderweele et al. (2020) recently proposed a flourishing measure aligned with the World Health Organization’s (WHO) health definition (World Health Organization [WHO], 1948; Vanderweele, 2017). Their measure encompasses six domains: happiness and life satisfaction, mental and physical health, meaning and purpose, character and virtue, intimate social relationships, and economic and material stability. This measure extends beyond psychological aspects, including physical health and socioeconomic status, which is particularly relevant as it underpins the maintenance of the other domains (Vanderweele, 2017). Therefore, the use of the flourishing index is a novel and promising approach to assessing and promoting well-being from multiple perspectives, and identifying the risk and promoting factors is important.

Previous research has shown that family, work, education, and religious communities as determinants of flourishing (Vanderweele, 2017); however, much research was exclusively done in Western countries (Tani et al., 2023). Considering the potential differences in the factors influencing flourishing across a variety of cultures, studies outside of Western countries including Japan would contribute to further understanding of flourishing. With its unique cultural characteristics, Japan is better positioned as a study site of flourishing.

Its uniqueness was highlighted especially during the COVID-19 pandemic. Japanese society was severely affected by the COVID-19 pandemic as other countries were: it underwent temporary school closures from March 2020 (Kyodo News, 2020) and the first state of emergency in April (The Japan Times, 2020). Instead of strict policy measures, such as lockdowns and curfews with legal punishments and penalties, the Japanese government asked people to refrain from going out voluntarily, requested social distancing, and restricted movement crossing prefectures, resulting in major changes in people’s lives (Ministry Of Health Labour And Welfare, 2020; Ministry Of Internal Affairs And Communications, 2020). In Japan, compared to Western countries, these measures were supported by a cultural background that respects orders and emphasizes consideration for others but further led to peer pressure (Yan et al., 2020; Wright, 2021). While this cultural background plays a role in containing the COVID-19 cases without strict governmental intervention, it also endangered people in a severely stressed situation.

For example, strong fear and anger arose against visitors from outside of Japan, and rule-breakers (Osaki, 2020) and people not wearing masks were attacked (Dukes, 2021; Ministry Of Justice, 2022), These aggressive reactions caused the government to postpone the 2020 Tokyo Olympics (International Olympic Committee, 2021) and ban mass-gathering events (Ministry Of Internal Affairs And Communications, 2020). A false rumor easily spreads around and makes people feel fear and anxiety in their unpredictable daily lives, forcing them to behave irrationally as in the case of toilet paper shortage (Komori, 2020). As a consequence of these stressful lives, people experience worsening mental health (Nagasu et al., 2021; Yamamoto et al., 2022), decreased physical activities (Otaki et al., 2022), social isolation (Murayama et al., 2021), binge drinking (Stickley et al., 2022), and increased suicidality (Sakamoto et al., 2021; Tanaka and Okamoto, 2021; Ueda et al., 2021). Flourishing is also damaged and exploring the mitigating factor is urgent.

According to an international comparative study on time use compiled by the Organization for Economic Cooperation and Development (OECD) in 2022, Japanese adult males work an average of 452 min per day, American males 320 min, and German males 290 min. Japanese adult women work an average of 272 min per day, compared to an average of 246 min per day for American women and 205 min per day for German women. Also, according to a 2015 OECD study, Japanese spend an average of 50 min commuting to work, Americans an average of 25 min, and Germans 45 min, indicating that Japanese spend more time on work-related activities. These results suggest that Japanese adults are more likely to be sleep-deprived than adults in comparable countries because of their longer work-related time. In a large-scale survey of U.S. citizens on their daily lifetime, the strongest association with shorter sleep was longer work hours, followed by longer commuting time (Basner et al., 2007). Furthermore, the OECD survey found that the average sleep duration of Japanese people was the second worst (OECD, 2011). This result indicates that Japanese adults are more likely to be sleep-deprived because they work longer hours than adults in comparable countries.

In addition, drinking and smoking habits were also specific in Japan. As for drinking, in a survey of per capita alcohol consumption in OECD countries, the average amount was 9.1 liters per capita, but the Japanese consumed 7.2 liters per capita, ranking 31st among the 40 OECD countries. However, the amount of alcohol consumed by the top 20% of the population accounted for approximately 70% of domestic alcohol consumption. This is more than in France, where the top 20% drink 50% of domestic alcohol consumption (11.8 liters per capita), or in the United Kingdom, where the top 20% drink 63% of domestic alcohol consumption (10.6 liters per capita). So in Japan, drinking was concentrated in a specific group of people (OECD, 2021).

About smoking, 27% of Japanese men smoke on a daily basis, ranking 7th among the 40 OECD countries. In contrast, the female smoking rate in the OECD is only 8%, ranking 3rd from the bottom; the average smoking rate in OECD countries is 20% for men and 13% for women, respectively. Smoking rates themselves, as in many OECD countries, have declined significantly over the past decade. However, restrictions on cigarette sales and rule of passive smoking are relatively less strict in Japan. Separation areas for smokers and non-smokers indoor and outdoor smoking is usual. Therefore, it is easier to smoke in Japan than in other countries (Rie Fujisawa, 2021).

In a study of children aged 17 years or younger, Tsao et al. (2021) found that children who had insufficient sleep tended to have a lower flourishing index than children who had sufficient sleep, specifically, a lower level of curiosity and restlessness. Reardon et al. (2023) reported that the flourishing score tends to be higher in adolescent students who sleep longer. In addition, Armand (Armand et al., 2021) in a report on college students and Richter (2015) in a report on adults also report that sleep duration is positively correlated with psychological well-being. Furthermore, Fuligni and Hardway (2006) in a study of adolescent students reported that short sleep duration decreased positive mood. Previous studies also examined association of drinking and smoking with flourishing; however, the results were mixed (Ohtani, 2012; Stickley et al., 2015; Dunbar et al., 2017; Parackal, 2017).

The purpose of this study is to investigate the association between lifestyle including sleep duration, drinking, and smoking, and flourishing index under the COVID-19 pandemic. We hypothesized that appropriate sleep duration, occasional drinking, and smoking, may be associated with flourishing during COVID-19. This is because, appropriate sleep duration is known as protective factor for mental illness (Zhai et al., 2015; Baglioni et al., 2016; Freeman et al., 2020; Schäfer et al., 2022), and occasional drinking is associated with social activities, which is crucial for well-being (Dunbar et al., 2017; Parackal, 2017), but smoking may not necessarily associate with social activities and induce further stress due to dependency (Stickley et al., 2015).

## 2 Materials and methods

### 2.1 Study participants

Data were obtained from the Utsunomiya COVID-19 seROprevalence Neighborhood Association (U-CORONA) study, a population-based study that aimed to evaluate the seroprevalence of COVID-19 in Utsunomiya City, Japan (Koyama et al., 2021; Nawa et al., 2021). In this study, self-report questionnaires were distributed via postal mail to 2295 people in 1000 households randomly selected from the Utsunomiya City Basic Resident Registry in November 2021 and collected at the survey site, along with written informed consent. Of the 2295 people, 1977 adults aged 18 years or older were the target population. A total of 484 adults participated in this study and completed the questionnaire.

For the analysis, 473 participants were included, excluding those with missing items in the flourishing questionnaire (*n* = 11). The study was conducted following the Declaration of Helsinki and was approved by the Ethics Committee of Tokyo Medical and Dental University (approval no. M2019-357).

### 2.2 Lifestyle

#### 2.2.1 Sleep duration

Sleep duration at the time of the survey was investigated with a questionnaire. The respondents were asked to select their sleep duration on weekdays from the following four options: <6 h, 6–8 h, 8–10 h, and >10 h.

According to the “Sleep Guidelines for Health Promotion 2014” by Japan’s Ministry of Health, Labor and Welfare, approximately 60% of Japanese adults sleep between 6 and 8 h. (Ministry Of Health Labour And Welfare, 2014). One study showed that male adult sleeps 6–8 h on average (Ohayon et al., 2004). Other cross-sectional studies have shown that sleeping less than 6 h led to excessive sleepiness during the day (Doi and Minowa, 2003; Ohayon et al., 2004). In addition, a survey study of 2,282 Japanese male workers followed for 14 years reported that those who slept less than 6 h per day had a 4.95-fold increased risk of developing myocardial infarction, angina pectoris, and other cardiovascular events compared to those who slept from 6–8 h (Hamazaki et al., 2011). Based on these previous reports, sleep duration was categorized into three groups (<6 h as the short sleep group, 6–8 h as the intermediate sleep group, and ≥ 8 h as the long sleep group).

#### 2.2.2 Drinking

For alcohol consumption, the respondents were divided into four groups: never/almost never drink, once to several times a month, once to several times a week, and once to several times a day.

#### 2.2.3 Smoking

Smoking was divided into three groups: never smokers, former smokers who had quit, and current smokers.

### 2.3 Flourishing index

Flourishing index was assessed using a self-reported questionnaire. This study used the flourishing measure developed by Vanderweele (2017) and Vanderweele et al. (2019). This measure consists of six domains: happiness and life satisfaction, mental and physical health, meaning and purpose, character and virtue, close social relationships, and financial and material stability (Vanderweele, 2017). As shown in Table 1, each domain was assessed using two questions (for example, the meaning and purpose domain includes “Overall, to what extent do you feel the things you do in your life are worthwhile?” and “I understand my purpose in life”). Each question was evaluated using an 11-point Likert scale from 0 to 10, with 10 indicating that the respondents perceived themselves positively; that is, a higher score indicated greater well-being. Domain-specific scores were calculated as the average of the two items in each domain. The flourishing index was computed as the average of the first five domains, and the secure flourishing index is the average of all domains (Vanderweele, 2017; Wȩziak-Białowolska et al., 2019). This flourishing measure has been validated and used in five culturally distinct countries, including the U.S., Sri Lanka, Cambodia, China, and Mexico (Wȩziak-Białowolska et al., 2019). In this study, the measure was translated into Japanese. Cronbach’s α for the flourishing index was 0.93, and the secure flourishing index was 0.92.

**TABLE 1:** Demographic characteristics of study participants (*n* = 473).

		Total (*N* = 473)	
		n	%
Sex	Male	223	47.1%
	Female	250	52.9%
Age (years)	18–40	113	23.9%
	40–64	206	43.6%
	≥65	154	32.6%
Household education level	Junior high/high school	194	41.0%
	Vocational	110	23.3%
	University/graduate school	166	35.1%
	Other, missing	3	0.6%
Household income (Japanese yen)	0– < 3M	120	25.4%
	3– < 6M	134	28.3%
	6–<10M	141	29.8%
	≥10M	56	11.8%
	Missing	22	4.7%
Sleeping hours (hours)	0– < 6 h	134	28.3%
	6– < 8 h	299	63.2%
	≥8 h	40	8.5%
Drinking	Almost never	235	49.7%
	Once to several times/month	42	8.9%
	Once to several times/week	107	22.6%
	Once to several times/day	89	18.8%
Smoking	Never smoking	306	64.5%
	Past smoking	110	23.3%
	Current smoking	57	12.2%

### 2.4 Covariates

Sex was investigated with a questionnaire. The respondents were asked to select their sex from the following two options: male and female. Sex was categorized into two groups (male group and female group). Age was investigated with a questionnaire. The respondents were asked their age by the following question: How old are you? And age was categorized into three groups (18–39 years old group, 40–64 years old, and ≥65 years old group). Annual income was investigated with a questionnaire. The respondents were asked their annual income by the following twelve options: 1. <JPY 1 million, 2. JPY 1-2 million, 3. JPY 2-3 million, 4. JPY 3-4 million, 5. JPY 4-5 million, 6. JPY 5-6 million, 7. JPY 6-7 million, 8. JPY 7-8 million, 9. 8-9 million, 10. JPY 9-10 million, 11. JPY 10 + million, 12. Missing. Annual income was categorized into five groups (less than JPY 3 million group, JPY 3–6 million group, JPY 6–10 million group, more than JPY 10 million group, and missing group).

### 2.5 Statistical analysis

All analyses were performed using Stata version 15 (Stata Statistical Software Release 15; College Station, TX, USA). We constructed univariate linear regression models. Flourishing score was regressed over sex, age, household education level, household income, smoking habits, drinking habits, and sleep duration as a separate model. In addition, we included all the covariates into one model, running the multivariate linear regression to explore the unique role of sleep duration while conditioning on the other demographic factors. Statistical significance was set at α < 0.05.

## 3 Results

With regard to sleep duration, we observed significantly lower levels of flourishing index in the short-sleep group compared with in the intermediate-sleep group (coef = −0.49, SE = 0.17, *p* < 0.01). No significant differences were observed in the levels of flourishing index between those in the long-sleep and intermediate-sleep groups (coef = −0.22, SE = 0.26, *p* = 0.41). Regarding alcohol consumption and smoking habits as covariates, the Flourishing index was significantly higher in the group that drank once to several times a week compared with the group that rarely drank (coef = 0.57, SE = 0.19, *p* < 0.01). There were no significant differences between the groups with respect to smoking (Table 2).

**TABLE 2:** Association between sleeping hours and flourishing (*N* = 473).

		Flourishing index					
		Univariate model			Multivariate model		
		coef	se	*p*-value	coef	se	*p*-value
Sleeping hours (ref: 6–<8 h)	<6 h	-**0**.**40**	0.17	**0**.**02**	-**0**.**49**	0.17	<**0**.**01**
	≥8 h	-0.96	0.27	0.72	-0.22	0.26	0.41
Drinking (ref: almost never)	Once to several times/month	0.40	0.26	0.13	0.48	0.27	0.07
	Once to several times/week	**0**.**47**	0.18	**0**.**01**	**0**.**57**	0.19	<**0**.**01**
	Once to several times/day	0.18	0.20	0.36	0.23	0.21	0.28
Smoking (ref: never smoking)	Past smoking	0.12	0.18	0.51	0.19	0.19	0.73
	Current smoking	-0.42	0.23	0.07	**0.24**	0.24	0.30
Sex (ref: male)	Female	0.18	0.15	0.21	0.36	0.17	**0**.**04**
Age (years) (ref: 41–64)	18–40	0.10	0.18	0.96	-0.09	0.19	0.64
	65≦	**0**.**64**	0.17	<**0**.**01**	**0**.**85**	0.19	<**0**.**01**
Education level (ref: university)	Junior high/high school	-0.21	0.17	0.21	-0.35	0.18	0.06
	Vocational	-0.06	0.19	0.77	-0.12	0.21	0.56
	Other/missing	-0.53	0.93	0.57	-0.44	0.89	0.62
Household Income (ref: 6–10M)	<3M	0.04	0.20	0.86	-0.29	0.21	0.17
	<6M	0.15	0.19	0.43	0.10	0.19	0.59
	≥10M	0.49	0.25	0.05	**0**.**55**	0.25	**0**.**03**
	Missing	-0.23	0.36	0.52	-0.40	0.35	0.26

Bold text indicates a significance level below the 0.05 significance level.

Multiple regression analysis of sleep duration and each domain of Flourishing index revealed significant differences in three of the five domains: Domain 1 (happy and life satisfaction), Domain 2 (mental and physical health), and Domain 3 (meaning and purpose). The scores for Domains 1, 2, and 3 were significantly lower in the short-time sleep group than in the normal-time sleep group (Domain 1: coef = −0.53, SE = 0.20, *p* = 0.01; Domain 2: coef = −0.53, SE = 0.20, *p* < 0.01; Domain 3: coef = −0.39, SE = 0.20, *p* = 0.04; Table 3).

**TABLE 3:** Association between sleeping hours and each domain of the Flourishing Index (*N* = 473).

		Happy and life satisfaction			Mental and physical health			Meaning and purpose			Character and virtue			Close social relationships		
		coef	se	p-value	coef	se	p-value	coef	se	p-value	coef	se	p-value	coef	se	p-value
Sleeping hours	<6 h	-**0**.**53**	0.20	**0**.**01**	-**0**.**53**	0.20	<**0**.**01**	-**0**.**39**	0.20	**0**.**04**	-0.25	0.19	0.18	-0.31	0.20	0.12
(ref: 6–<8 h)	≥8 h	0.09	0.33	0.78	-0.16	0.32	0.63	-0.11	0.32	0.73	-0.08	0.30	0.80	-0.24	0.32	0.45

Bold text indicates a significance level below the 0.05 significance level.

Multiple regression analysis of drinking and each domain of Flourishing index revealed significant differences in two of the five domains: Domain 1 (happy and life satisfaction), Domain 2 (mental and physical health). The scores for Domains 1 and 2 were significantly higher in the group that drank once to several times a week compared with the group that rarely drank (Domain 1: coef = 0.65, SE = 0.23, *p* < 0.01; Domain 2: coef = −0.67, SE = 0.22, *p* < 0.01; Table 4).

**TABLE 4:** Association between drinking and each domain of the Flourishing Index (*N* = 473).

		Happy and life satisfaction			Mental and physical health			Meaning and purpose			Character and virtue			Close social relationships		
		coef	se	p-value	coef	se	p-value	coef	se	p-value	coef	se	p-value	coef	se	p-value
Drinking	Once to several times/month	**0**.**68**	0.33	**0**.**04**	**0**.**76**	0.32	**0**.**02**	0.34	0.32	0.28	-0.55	0.30	0.86	0.24	0.32	0.45
(ref:almost never)	Onece to several times/week	**0**.**65**	0.23	<**0**.**01**	**0**.**67**	0.22	<**0**.**01**	0.36	0.22	0.09	0.19	0.21	0.36	0.34	0.22	0.12
	Once to several times/day	0.44	0.24	0.07	**0**.**53**	0.23	**0**.**02**	0.08	0.23	0.73	-0.08	0.22	0.71	-0.13	0.23	0.59

Bold text indicates a significance level below the 0.05 significance level.

Multiple regression analysis of smoking and each domain of Flourishing index revealed significant differences in two of the five domains: Domain 1 (happy and life satisfaction), Domain 3 (meaning and purpose). The scores for Domains 1 and 3 were significantly lower among the current smoking group compared with the never smoking group (Domain 1: coef = −0.67, SE = 0.28, *p* = 0.02; Domain 3: coef = −0.70, SE = 0.27, *p* < 0.01; Table 5).

**TABLE 5:** Association between smoking and each domain of the Flourishing Index (*N* = 473).

		Happy and life satisfaction			Mental and physical health			Meaning and purpose			Character and virtue			Close social relationships		
		coef	se	p-value	coef	se	p-value	coef	se	p-value	coef	se	p-value	coef	se	p-value
Smoking	Past smoking	0.33	0.22	0.14	0.03	0.21	0.88	0.11	0.21	0.60	0.08	0.20	0.70	0.03	0.21	0.89
(ref:never smoking)	Current smoking	-**0**.**67**	0.28	**0**.**02**	-0.51	0.27	0.06	-**0**.**70**	0.27	**0**.**01**	-0.22	0.26	0.40	-0.15	0.28	0.59

Bold text indicates a significance level below the 0.05 significance level.

## 4 Discussion

In this study, we aimed to investigate the association between lifestyle and the flourishing under stressful conditions, such as the COVID-19 pandemic. We found that appropriate sleep duration and occasional drinking was associated with flourishing, but not for smoking.

Interestingly, several demographics showed significant association with flourishing. Female showed a higher score of flourishing during the COVID-19 pandemic (coef = 0.36, SE = 0.17, *p* = 0.04; Table 2). One possible reason for the sex difference may be changes in the work environment during the COVID-19 pandemic. According to a national panel survey in Japan, half of the employees expected a decrease in future income during the COVID-19 pandemic and were anxious about surviving in society (Enatsu, 2021). Since most males in the present study were employed, males may be more negatively affected by job insecurity and deprivation of social support at the workplace.

In addition, older people showed higher flourishing score (coef = 0.85, SE = 0.19, *p* < 0.01; Table 2), even during COVID-19 pandemic, which is more threatened for older people. This difference by age is consistent with previous studies showing older adults to have higher psychological well-being than younger adults (Stone et al., 2010; Momtaz et al., 2014). This may be because older adults have greater ability to control bad moods than young adults, as reported by Carstensen et al. (2000). Furthermore, social restrictions, such as curfews, were reported to have a stronger impact on young adults, and the magnitude of stress during a pandemic tended to be greater in younger age groups (Lee et al., 2020; Smith et al., 2020; Coppola et al., 2021; Royalty Marketing Inc., 2022). Even discounting the fact that older adults have higher coronary morbidity and mortality rates than younger adults, these social restrictions might have more strongly affected the flourishing score.

As for income, only the group with an annual income of over 10 million yen had a significantly higher flourishing score than the group with an annual income of than 6–10 million yen (coef = 0.55, SE = 0.25, *p* = 0.03; Table 2). According to Killingsworth (2021), happiness tends to increase as income increases up to an annual income of seven million yen.

This study confirmed the hypothesis that sleep duration is significantly associated with the levels of flourishing, showing that sleep duration <6 h increased the risk of lower levels of flourishing. For each of the domains of Flourishing index and sleep duration, there was no significant difference in the domains for the longer sleep duration group compared with the moderate sleep duration group. However, the short sleep group scored significantly lower on happiness and satisfaction, mental and physical health, and meaning and purpose domains. The results for happiness and mental and physical health domains were significantly lower in the short sleep duration group but not in the long sleep duration group. Previous studies on sleep duration and quality of life (QOL) have reported associations between sleep duration and physical and mental QOL (Magee et al., 2011; Chen et al., 2014; Cohrdes et al., 2018; Matsui et al., 2020), whereas some reported that sleep duration had little effect on physical QOL (Magee et al., 2011; Ge et al., 2019). The importance of sleep duration with respect to reduced QOL has been well-documented (Kim et al., 2015; Cohrdes et al., 2018; Lallukka et al., 2018; Matsui et al., 2020). Although the meaning and purpose domain is not included in the index for determining QOL and cannot be examined in relation to previous papers, mental health may also affect meaning and purpose, necessitating further studies. Nevertheless, our finding suggests that, under the intense stress of the COVID-19 pandemic, short sleep duration may decrease multidimensions of well-being. This study also added an important insight as the role of sleep duration may extend to flourishing to this accumulating literature on sleep and QOL.

The insignificant difference in flourishing between participants in the long-sleep and intermediate-sleep groups was also an important finding. Previous studies have found that both short and long sleep durations were associated with an increased risk of adverse health outcomes (Patel et al., 2004; Tamakoshi and Ohno, 2004) In addition, an inverse U-shaped association between sleep duration and QOL was reported (Knutson and Turek, 2006; Kim et al., 2015). However, another demonstrated that prolonged sleep does not affect QOL (Matsui et al., 2020); long sleep might be a consequence of unrecognized comorbidities, which in turn could induce QOL deterioration (Stranges et al., 2008; Matsui et al., 2020). Considering the small sample size in the long-sleep group in the current analysis, the missing of significant differences was attributable to the lack of power or chance findings, and our findings provide limited implications regarding longer sleep duration. Further studies with large sample sizes are warranted.

The Flourishing index was significantly higher in the group that drank once to several times a week compared with the group that did not drink at all. These results are in line with reports by Ohtani (2012) and Parackal (2017) who reported that moderate alcohol consumption increases life happiness. Although recent study found the adverse effect of drinking in the lower amount for longevity (Topiwala et al., 2017), there might be positive effect of occasional drinking for flourishing, especially during pandemic, when the social events are limited.

The results of this study were surprising because in previous reports, smokers have reported lower levels of happiness than non-smokers (Taylor et al., 2014; Wang et al., 2014; Stickley et al., 2015). Smokers scored significantly lower on domains 1 (happiness and life satisfaction) and domain 3 (meaning and purpose), and there were no significant differences in scores in the other domains, but scores tended to decrease in the mean. However, as for the lack of significant differences overall, this may be due to the special environment of the pandemic. The high level of stress placed on everyone during the pandemic resulted in an overall tendency toward depression and decreased vitality. It was inferred that the greater impact of economic burden on the Flourishing index diluted the impact of smoking on the Flourishing index. However, further research is needed on the incidence and severity of depression in smokers and non-smokers during pandemics and normal times.

This study had some limitations. This was a cross-sectional research design, in which causality could not be established. In addition, because the questionnaire did not include the assessment of sleep quality, the association between sleep quality and flourishing index was left for future studies. While we assessed sleep duration via a self-reported questionnaire, future research might be better to use experimental or longitudinal designs to examine the associations between sleep quality, sleep duration, and QOL. Moreover, although our survey during the COVID-19 pandemic provides unique insight into flourishing under strong social stress, this limits the generalizability. Therefore, it would be desirable to conduct a similar study and compare it with other stressful situations or non-stressful conditions.

## 5 Conclusion

Adequate sleep duration and occasional drinking maybe important for flourishing during COVID-19. Therefore, ensuring adequate sleep without cutting down on sleep time, and occasional drinking may contribute to a better life, especially in a highly stressful situation, such as a pandemic.

## Data availability statement

The datasets presented in this article are not readily available because the ethics committee did not approve data sharing. Requests to access the datasets should be directed to TF, fujiwara.hlth@tmd.ac.jp.

## Ethics statement

The studies involving humans were approved by the Ethics Committee at Tokyo Medical and Dental University. The studies were conducted in accordance with the local legislation and institutional requirements. The participants provided their written informed consent to participate in this study.

## Author contributions

TS: Conceptualization, Formal analysis, Writing – original draft. YY: Conceptualization, Data curation, Formal analysis, Supervision, Writing – review and editing. NN: Conceptualization, Data curation, Formal analysis, Supervision, Writing – review and editing. HN: Data curation, Writing – review and editing. YK: Data curation, Writing – review and editing. JK: Data curation, Writing – review and editing. TF: Conceptualization, Data curation, Funding acquisition, Supervision, Writing – review and editing.
